# Emerging carbapenem-resistant *Klebsiella pneumoniae* sequence type 16 causing multiple outbreaks in a tertiary hospital in southern Vietnam

**DOI:** 10.1099/mgen.0.000519

**Published:** 2021-02-10

**Authors:** To Nguyen Thi Nguyen, Phuong Luong Nha Nguyen, Ngan Thi Quynh Le, Lan Phu Huong Nguyen, Thuy Bich Duong, Nghia Dang Trung Ho, Quynh Pham Nhu Nguyen, Trung Duc Pham, Anh Tuan Tran, Hao Chung The, Hien Huu Nguyen, Chau Van Vinh Nguyen, Guy E. Thwaites, Maia A. Rabaa, Duy Thanh Pham

**Affiliations:** ^1^​ Oxford University Clinical Research Unit, Ho Chi Minh City, Vietnam; ^2^​ Hospital for Tropical Diseases, Ho Chi Minh City, Vietnam; ^3^​ Pham Ngoc Thach University of Medicine, Ho Chi Minh City, Vietnam; ^4^​ Centre for Tropical Medicine and Global Health, University of Oxford, Oxford, UK

**Keywords:** carbapenem resistance, colistin resistance, extensive drug resistance, *Klebsiella pneumoniae*, nosocomial outbreaks, ST16

## Abstract

The emergence of carbapenem resistance in *Klebsiella pneumoniae* represents a major global public health concern. Nosocomial outbreaks caused by multidrug-resistant *K. pneumoniae* are commonly reported to result in high morbidity and mortality due to limited treatment options. Between October 2019 and January 2020, two concurrent high-mortality nosocomial outbreaks occurred in a referral hospital in Ho Chi Minh City, Vietnam. We performed genome sequencing and phylogenetic analysis of eight *K. pneumoniae* isolates from infected patients and two environmental isolates for outbreak investigation. We identified two outbreaks caused by two distinct lineages of the international sequence type (ST) 16 clone, which displayed extensive drug resistance, including resistance to carbapenem and colistin. Carbapenem-resistant ST16 outbreak strains clustered tightly with previously described ST16 *K. pneumoniae* from other hospitals in Vietnam, suggesting local persistence and transmission of this particular clone in this setting. We found environmental isolates from a hospital bed and blood pressure cuff that were genetically linked to an outbreak case cluster, confirming the potential of high-touch surfaces as sources for nosocomial spread of *K. pneumoniae *. Further, we found colistin resistance caused by disruption of the *mgrB* gene by an IS*L3*-like element, and carbapenem resistance mediated by a transferable IncF/*bla*_OXA-181_ plasmid carrying the IS*L3*-like element. Our study highlights the importance of coordinated efforts between clinical and molecular microbiologists and infection control teams to rapidly identify, investigate and contain nosocomial outbreaks. Routine surveillance with advanced sequencing technology should be implemented to strengthen hospital infection control and prevention measures.

## Data Summary

1. All supplementary data (Table S1, Figs S1–S3, available with the online version of this article) have been deposited in FigShare (10.6084/m9.figshare.13378745).
2. All short reads used in this study have been deposited in the European Nucleotide Archive (ENA) under study accession number PRJEB38898 (accession numbers: ERS4672941–ERS4672951).
3. The long reads from Nanopore sequencing have been deposited in the ENA (accession number: ERS4678223).
4. The plasmid sequence was deposited in GenBank (accession number: MT635909).

**Outcome**

Two independent high-mortality hospital-based outbreaks of carbapenem-resistant *Klebsiella pneumoniae* were identified and controlled in the Hospital for Tropical Disease in Ho Chi Minh City, Vietnam, through coordinated efforts between clinical microbiologists, molecular biologists and infection control teams in late 2019 and early 2020. Whole-genome sequencing and phylogenetic analysis represent a powerful tool enabling us to characterize and trace the transmission of nosocomial outbreaks in a timely manner.

## Introduction

The spread of multidrug-resistant Gram-negative bacteria is one of the most concerning issues in global health [1–3]. Among these, carbapenem-resistant *Enterobacteriaceae* have recently been singled out as an urgent threat to global public health by the U.S. Centers for Disease Control and Prevention (CDC), the World Health Organization (WHO) and Public Health England (PHE) [1, 4, 5]. In particular, *Klebsiella pneumoniae* that are resistant to last-line antimicrobials such as carbapenems, colistin and tigecycline have been increasingly reported, and pose substantial challenges for treatment [6, 7]. *K. pneumoniae* can colonize the gastrointestinal tract, skin and nasopharynx in humans, and cause both community-onset and hospital-onset infections. In the early 1970s, *K. pneumoniae* became an important cause of nosocomial infections, such as urinary tract infections, respiratory tract infections and bloodstream-associated infections (BSIs) [8]. Notably, drug-resistant *K. pneumoniae* is a common cause of nosocomial outbreaks worldwide, with particularly high mortality rates following the emergence of carbapenem resistance [9–15]. The mortality rates attributable to carbapenem-resistant *K. pneumoniae* generally range from 20 to 30 % and have reached up to 75 % in bloodstream infections among neonates [3, 14, 16, 17]. While nosocomial outbreaks caused by carbapenem-resistant *K. pneumoniae* often occur in intensive care units (ICUs), hospital-wide outbreaks are not uncommon [10].

The mechanisms of carbapenem resistance in *K. pneumoniae* primarily involve the production of β-lactamases combined with a decrease in outer membrane permeability and the production of true carbapenemases [18]. The most common carbapenemases in *K. pneumoniae* include the class A *K. pneumoniae* carbapenemases (KPCs), class B NDMs (New Delhi metallo-β-lactamases), and class D OXA-48 carbapenemase and its derivatives. Carbapenemase-producing *K. pneumoniae* emerged in different parts of the world in the late 1990s (KPCs) [19] and 2000s (OXA-48 and NDM-1) [20, 21], and subsequently spread globally. The KPC-producing *K. pneumoniae* are often associated with invasive high-risk clones such as sequence type (ST) 258 and ST11 [22], whereas NDM and OXA-48 carbapenemases are found across various *K. pneumoniae* clones [18]. KPC-producing ST258 and ST11 have now become endemic in the USA and many European and Asian countries, following their international dispersal in the mid-2000s [15, 23, 24]. The NDM-carbapenemase-producing *K. pneumoniae* are predominantly found in South Asian countries and sporadically reported in other countries around the world. OXA-48-producing *K. pneumoniae* are endemic in Turkey, North Africa and Europe, while their derivative, OXA-181, is more common in the Indian subcontinent [25]. The carbapenem-resistance genes are often mediated by mobile elements including plasmids and transposons, which can facilitate their spread across diverse members of the *Enterobacteriaceae* [26, 27].

Alternative last-line antimicrobials, such as colistin and tigecycline, are often used to treat carbapenem-resistant *K. pneumoniae* infections [16]. Colistin is a cationic antimicrobial that targets the anionic lipid A phosphate moiety of bacterial lipopolysaccharide (LPS) to break the cell membrane and cause cell death [28]. However, resistance to colistin has been increasingly reported in recent years; for example, 17 and 43 % of carbapenem-resistant *K. pneumoniae* in Taiwan (2010–2012) and Italy (2013–2014), respectively, were also found to be colistin resistant [29, 30]. The primary mechanisms of colistin resistance involve modifications of the bacterial LPS caused by mutations in two-component systems (TCSs) such as *pmrA/B*, *phoQ/P* and *crrA/B*, as well as mutations/disruptions of the *mgrB* gene (TCS regulator). Mutations occurring in the TCSs result in up-regulation of the *pmrABC* and *pmrFHIJKLM* operons and the *pmrE* gene, which subsequently leads to LPS modification and colistin resistance [31–34]. Additionally, mutations/disruptions of the *mgrB* gene, which exerts negative feedback on PhoQ/P, leads to the up-regulation of PhoQ/P, followed by activation of the *pmrFHIJKLM* operon and colistin resistance [35, 36]. Plasmid-mediated colistin resistance has also been reported in *K. pneumoniae* strains of both human and animal origin in China [37, 38].

In Vietnam, there are limited epidemiological data to help understand hospital-acquired infections (HAIs); however, some studies have shown that *K. pneumoniae* is one of the most common pathogens causing HAIs in the country, especially in ICUs, with increasing prevalence of carbapenem resistance over time [39–41]. From October 2019 to January 2020, two distinctive nosocomial outbreaks with high mortality caused by carbapenem-resistant *K. pneumoniae* occurred in two separate wards of a large referral hospital in Ho Chi Minh City, Vietnam. In this study, we aimed to describe the clinical manifestations and investigate the molecular epidemiology of these *K. pneumoniae* outbreak strains.

## Methods

### Description of outbreaks in the Hospital for Tropical Diseases, Ho Chi Minh City, Vietnam

The Hospital for Tropical Diseases in Ho Chi Minh City is a large referral hospital for infectious diseases in southern Vietnam. The hospital has about 550 beds and sees over 2500 outpatients every day. The majority of patients (70 %) are residents of Ho Chi Minh City, while the remaining patients come from the surrounding provinces. A previous hospital-based study of BSIs at the Hospital for Tropical Diseases between 2010 and 2014 showed that *K. pneumoniae* was the second most common causative agent of BSIs, though carbapenem resistance in *K. pneumoniae* was uncommon during this time period (5.7  % in 2014) [42]. Between October 2019 and January 2020, the microbiology department of the Hospital for Tropical Diseases noted an increase in nosocomial infections caused by carbapenem-resistant *K. pneumoniae* in two separate wards, the adult intensive care unit (AICU) and the Viet-Anh (VA) ward (which cares for patients with central nervous system infections). The antimicrobial resistance (AMR) profiles of carbapenem-resistant *K. pneumoniae* isolates from the AICU and the VA ward were distinct, suggesting the emergence of two nearly simultaneous nosocomial outbreaks.

The outbreak in the AICU (referred to as outbreak one) occurred between October and December 2019, and involved three patients from whom carbapenem- and colistin-resistant *K. pneumoniae* isolates were recovered from bronchoalveolar lavage (BAL) and blood samples. The outbreak on the VA ward (referred to as outbreak two) occurred between November 2019 and January 2020, and involved five patients; carbapenem-resistant *K. pneumoniae* isolates were identified from urine, BAL, sputum, pus and blood samples. Both the AICU and VA ward are well practiced in intensive care treatment and invasive procedures, and are located in separate hospital buildings with distinct clinical staff.

### Hospital infection-control measures

In response to notification from the microbiology department regarding the *K. pneumoniae* outbreaks, the hospital infection-control team conducted a number of rigorous measures to identify the sources and contain the spread of these organisms. These measures included enhanced environmental sampling (patient monitors, haemodialysis machines, medical trolleys, bedside items, bedrails, ventilators, infusion pumps, stethoscopes, blood-pressure cuffs, suction catheters and sinks), informing the healthcare workers (clinicians, nurses, housekeepers) about the infected *K. pneumoniae* patients, isolating the patients in private rooms with dedicated nurses and non-critical patient-care equipment (stethoscopes, blood pressure cuffs, thermometers), cleaning all surfaces in the rooms (shared and dedicated equipment, beds, trolleys, door handles, toilets, etc.) with bleach three times per day, strict enforcement of hand hygiene and universal use of gowns and gloves for all healthcare personnel before entering and upon leaving patient rooms, decontamination of all rooms and equipment used by the *K. pneumoniae *-infected patients with hydrogen peroxide vapour after discharge, and enhanced cleaning and handwashing within the AICU and VA ward.

During the outbreaks, 56 and 48 environmental samples were collected by extensive sampling on high-touch surfaces in the AICU and VA ward, respectively. Four carbapenem- and colistin-resistant *K. pneumoniae* isolates were identified from a patient monitor, ventilator, medical trolley and bed rail in the AICU; however, these isolates were not stored and were unavailable for whole-genome sequencing (WGS). In addition, two carbapenem-resistant *K. pneumoniae* isolates were recovered from a bedrail and a blood pressure cuff in the VA ward. These two isolates were subsequently genome sequenced and compared to outbreak strains.

### Bacterial identification and antimicrobial profiles

The *K. pneumoniae* isolates (Table S1) were identified from different types of samples (BAL, urine, blood, pus and sputum) collected during the two outbreaks following routine hospital procedures. In brief, blood culture was performed using an automatic instrumented culture system, BD BACTEC (Becton Dickinson) or BacT/Alert (bioMérieux). Positive samples flagged by the systems were sub-cultured onto different types of media (blood agar, MacConkey agar, Chromagar) and incubated at 37 °C for 24 h to isolate the infecting organisms. Other samples (BAL, urine, pus and sputum) were directly cultured onto agar plates as per routine microbiological procedures. *K. pneumoniae* was identified using MALDI-TOF MS (Bruker Daltonics).

The antimicrobial susceptibility of collected isolates was assessed using the VITEK2 system (bioMérieux), and Clinical and Laboratory Standards Institute (CLSI) guidelines were applied to identify antimicrobial susceptibility breakpoints [43]. The antimicrobials used for *K. pneumoniae* susceptibility testing included amikacin, cefepime, ceftazidime, ciprofloxacin, colistin, gentamicin, imipenem, levofloxacin, meropenem, piperacillin/tazobactam, rifampicin, ticarcillin/clavulanic acid and trimethoprim/sulfamethoxazole. Additionally, minimal inhibitory concentrations (MICs) against colistin were measured by Etest (AB Biodisk) on Mueller–Hinton agar according to the manufacturer’s instructions, and susceptibility MIC breakpoints were interpreted using CLSI guidelines [43].

### WGS

A total of ten isolates (three from outbreak one, five from outbreak two and two from environmental samples related to outbreak two) were subjected to DNA extraction using a Wizard genomic DNA extraction kit (Promega) [44]. A Qubit fluorometer was used for measuring DNA concentrations following DNA extraction. For WGS, 1 ng extracted DNA from each strain was used to create a sequencing library using the Nextera XT library preparation kit, as per the manufacturer’s instructions. WGS was performed on an Illumina MiSeq platform to generate 250 bp paired-end reads [45]. Raw reads were deposited in the European Nucleotide Archive (ENA) under study accession number PRJEB38898 (Table S1).

### SNP calling and phylogenetic analyses

The FastQC and FASTX-ToolKit bioinformatics tools were used to assess the quality of raw reads and for removal of adapters and low-quality bases prior to downstream analysis [46, 47]. All raw reads that passed quality checking were then mapped to the reference genome MGH78578 (accession number: CP000647.1) using RedDog pipeline v1.10b, as described previously [48]. Briefly, RedDog used local sensitive mapping Bowtie2 to map all raw reads to the reference genome, and SNPs were identified using SAMtools v1.3.1 [49]. SNPs that did not meet the quality criteria (Phred score ≥30, depth coverage ≥5) were excluded from the analysis. The sequences failed mapping when <50 % of reads mapped to the reference genome, the ratio of heterozygous/homozygous exceeded 20 % or the total assembly length was outside the expected range (5–6.5 Mb) [50, 51]. Finally, a concatenation of 12 190 SNPs that were present in more than 95 % of all genomes was utilized for phylogenetic analysis. RAxML (Randomized Axelerated Maximum Likelihood) [52] was used to reconstruct a maximum-likelihood (ML) phylogenetic tree from the above alignment using the generalized time-reversible evolutionary model with gamma-distributed rate variation (GTR**+**G). One hundred bootstrap pseudo-replicate analyses were performed to assess the robustness of the ML tree topology [52].

To investigate the evolutionary histories of *K. pneumoniae* ST16 isolates in a larger context, genomic data from all nine ST16 isolates from this study were combined with raw reads from 36 ST16 genomes obtained from GenBank. SNP calling was performed using the RedDog pipeline as described above with the same reference genome (MGH78578) to generate a pseudogenome alignment. This was subsequently subjected to the spatial scanning statistic using Gubbins [53] to identify recombinant regions. SNPs identified in recombinant regions were subsequently removed, resulting in a final alignment length of 826 SNPs. RAxML was used to reconstruct the ML phylogeny from this alignment using the GTR+G nucleotide substitution model. An ST412 *K. pneumoniae* isolate was used as an outgroup to root this tree. Support for this phylogenetic tree was assessed via bootstrap analysis with 100 pseudo-replicates.

### Gene content

Known alleles of acquired resistance genes and virulence genes were directly detected from a read set mapping approach based on short read sequence typer SRST2 [54]. We used the ARG-Annot database for the detection of AMR genes and the PlasmidFinder database for plasmid multilocus sequence typing (MLST) [55]. The virulence genes and MLST were analysed using the *K. pneumoniae* BIGSdb database at the Institute Pasteur (http://bigsdb.web.pasteur.fr). All raw reads were *de novo* assembled using Unicycler v0.4.8 to generate the contigs [56]. The structures of capsule types, O antigens and siderophores were predicted from all genome assemblies using Kleborate v0.4.1 (https://github.com/katholt/Kleborate).

### Nanopore sequencing

One colistin-resistant *K. pneumoniae* isolate from outbreak one was subjected to long-read Nanopore sequencing to characterize the colistin resistance mechanism and resolve the carbapenem resistance plasmid structure. Genomic DNA was extracted from the colistin-resistant *K. pneumoniae* strain isolated from patient P3_O1 (MIC for colistin was 8 µg ml^−1^), and the sequencing library was prepared using the rapid 1D sequencing kit SQK-RAD002 (Oxford Nanopore Technologies), according to the manufacturer’s recommendations. The MinION Mk1, FLO-MIN106 flow cell and MinKNOW software v1.13 were used for sequencing and base calling. Nanopore long reads obtained from Nanopore sequencing were combined with Illumina short-read data using Unicycler v0.4.8 to produce a hybrid assembly. The full-length plasmid produced was annotated using prokka v1.12 [57]. Raw MinION reads were deposited in the ENA (accession no. ERS4678223).

## Results

### Clinical descriptions of *K. pneumoniae* outbreaks

#### Outbreak one (AICU)

**Fig. 1.**
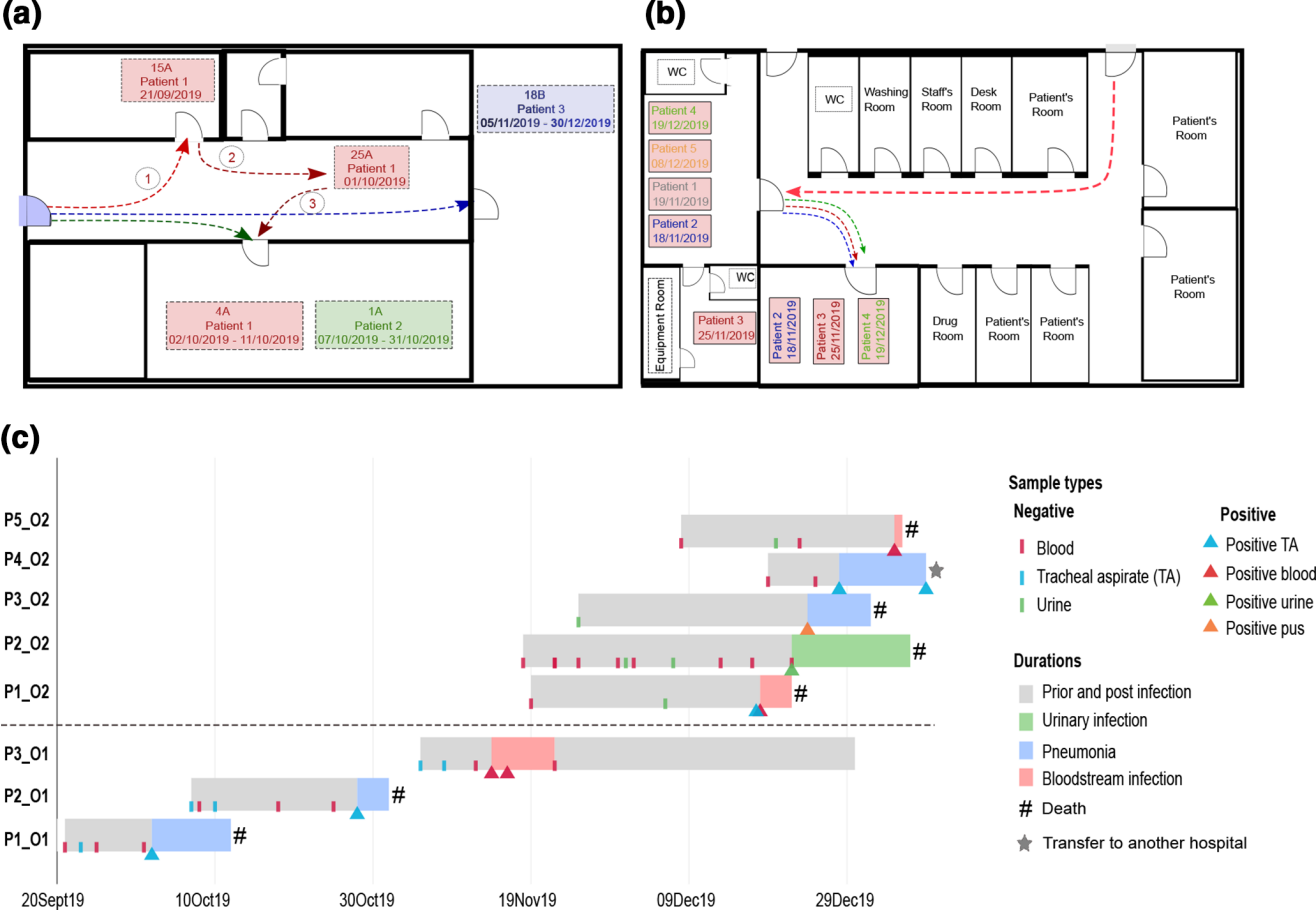
Hospital wards, patient movement and timeline of the *K. pneumoniae* outbreaks. (**a**) Map of the AICU and patient movement during outbreak one. The coloured arrows show the movement of the three patients. The circled numbers indicate the movement of patient one; bed numbers are shown in the map (i.e. 15A, 25A, 4A, 1A, 18B). (**b**) Map of the VA ward and circulation of patients during outbreak two. The coloured arrows show the movement of patients.** (c**) Timeline showing duration of hospital stay, diagnosis and outcome for the patients during the outbreaks in the AICU and VA ward. The dashed line separates patients from the two outbreaks [outbreak one (O1), bottom; outbreak two (O2), top].

The first case (P1_O1) was a 54-year-old female admitted to the AICU on 21st September 2019 with a diagnosis of cellulitis (Fig. 1a). The patient developed septic shock within hours of admission and was treated with broad-spectrum antimicrobials (imipenem and vancomycin). Invasive procedures including endotracheal intubation, mechanical ventilation, continuous renal replacement therapy, central venous catheterization, urinary catheterization and nasogastric intubation were also applied. After 2 days, the first tracheal aspirate (TA) culture was sterile and the patient’s health improved. Extubation was performed on 28th September, after which the patient’s condition worsened. Four days later, on 2nd October, the patient experienced sudden cardiac arrest requiring cardiopulmonary resuscitation. Culture of a TA sample on this day grew extensively drug resistant (XDR) *K. pneumoniae *, which was resistant to almost all antimicrobials including carbapenems and colistin, but remained susceptible to ceftazidime and gentamicin. Chest X-ray showed infiltrates in the lower right lobe and a diagnosis of pneumonia was made. Ceftazidime and colistin were added to the treatment on 7th October, after which the patient’s high fever subsided. Two days later, the patient suddenly developed severe upper gastrointestinal bleeding and experienced a second cardiac arrest event, leading to death after 15 days in hospital (Fig. 1c).

The second case (P2_O1) was a 61-year-old male patient with severe pneumonia who was admitted to the AICU on 7th October 2019 and was placed in the same room as P1_O1 (Fig. 1a). He experienced septic shock after 5 days (12th October), and invasive procedures similar to those used for P1_O1 were applied. Although two TA and three blood samples were initially found negative by culture, XDR *K. pneumoniae* was detected from a TA sample on 28th October; and the patient expired 3 days later due to severe sepsis from pneumonia.

On 5th November 2019, patient three (P3_O1), a 25-year-old female with severe dengue fever was admitted to the same room in which P1_O1 and P2_O1 had undergone treatment (Fig. 1a). P3_O1 was treated with continuous renal replacement therapy, an arterial catheter was inserted to facilitate frequent blood sampling and blood-pressure monitoring, and a central venous catheter was applied for monitoring central venous pressure and administration of medication. Blood cultures were sterile three times before culturing XDR *K. pneumoniae* on 14th November (Fig. 1c). P3_O1 slowly recovered from this infection and antimicrobial administration (meropenem, colistin, gentamicin and trimethoprim/sulfamethoxazole) was stopped on 28th November. She was discharged on 30th December.

#### Outbreak two (VA ward)

The first patient (P1_O2) was a 34-year-old male who was transferred to the VA ward from the HIV ward at the Hospital for Tropical Diseases and diagnosed with cryptococcal meningitis on 19th November 2019 (Fig. 1b). He was initially treated with amphotericin B and fluconazole. After 2 weeks of treatment, his cerebrospinal fluid culture was negative and treatment with amphotericin B was suspended. The patient was moved to ward C in this hospital, because he required less intensive observation (Fig. 1b). He developed high fever and respiratory failure 3 days later. The patient was diagnosed with severe pneumonia and treatment with piperacillin/tazobactam was initiated. The patient was moved back to the VA ward for intubation and mechanical ventilation on 7th December. Amphotericin B and dexamethasone were re-administered due to suspicion of worsening cryptococcal meningitis. After treatment with piperacillin/tazobactam for 7 days, the pneumonia was controlled and the fever subsided. On 15th December, the patient was again febrile and his white blood cell count was 20 000 cells µl^−1^ with a high neutrophil count (80 %) suggestive of a new infection. Blood and TA culture were performed on 18th December and meropenem was administered. The blood and TA were found positive for carbapenem-resistant *K. pneumoniae* on 20th December, and colistin was added to the treatment. The patient’s condition worsened, with septic shock, severe gastrointestinal bleeding and eventual death on 22nd December (Fig. 1c).

Patient two (P2_O2) was a 19-year-old female with encephalitis who had been admitted to another hospital for 2 days before being transferred to the VA ward at the Hospital for Tropical Diseases on 18th November 2019 (Fig. 1b). The patient was admitted to the hospital with a urinary catheter that had been administered at the previous hospital. On 22nd December, her urine culture was positive for carbapenem-resistant *K. pneumoniae *, for which treatment with amikacin was applied for 7 days. She was moved to another room for isolation (Fig. 1b). The patient expired on 6th January 2020 due to septic shock (Fig. 1c).

Patient three (P3_O2) was admitted on 25th November 2019 with suspected meningitis and carbapenem-resistant *K. pneumoniae* were isolated from pus from a tracheostomy site on 24th December. The patient died due to septic shock from pneumonia.

Patient four (P4_O2) was admitted on 19th December 2019 with suspected meningitis. Blood culture was performed on 19th and 25th December, and the results were negative. TA culture was positive for carbapenem-resistant *K. pneumoniae* on 28th December and again on 8th January 2020. Patient four was subsequently moved to another hospital for treatment of tuberculous meningitis, without resolution of the *K. pneumoniae* infection.

The final case in this outbreak (P5_O2) was a 42-year-old male with cryptococcal meningitis who was treated with amphotericin B, fluconazole and dexamethasone from the day of admission (8th December 2019). A central venous catheter was applied on 27th December. After 8 days, the patient experienced septic shock and was treated with imipenem. Blood culture was positive for *Escherichia coli* and carbapenem-resistant *K. pneumoniae* on 4th January 2020. The patient expired the following day (Fig. 1c).

### AMR of *K. pneumoniae* isolates from the two outbreaks and environmental samples

**Fig. 2.**
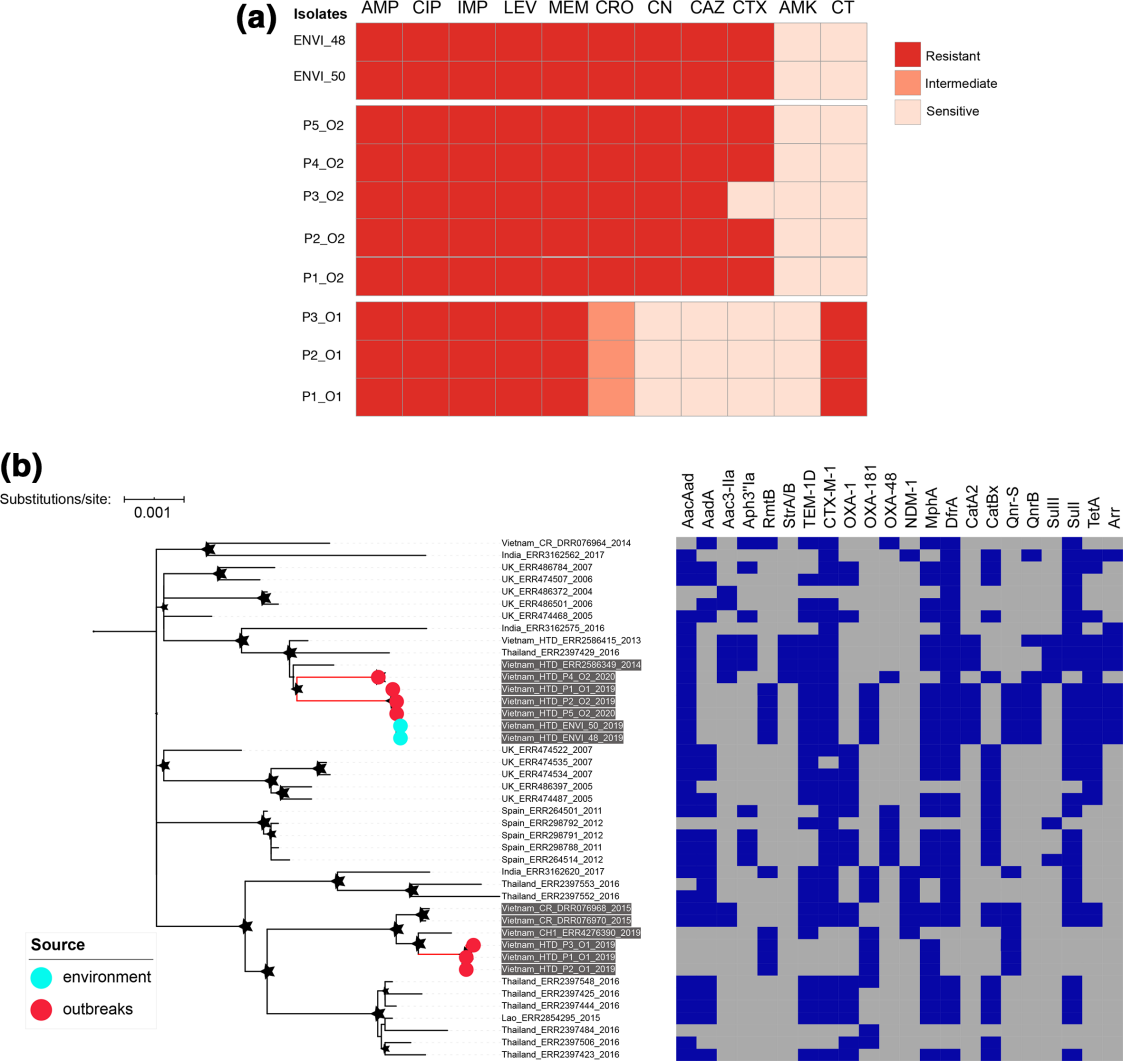
AMR phenotype and phylogeny of ST16 *K. pneumoniae* from the outbreaks and environmental samples. (**a**) Antimicrobial susceptibility of outbreak isolates (O1, outbreak one; O2, outbreak two) and environmental isolates (ENVI). Red, orange and light orange colours correspond to full resistance, intermediate resistance and sensitivity, respectively. AMP, Ampicillin; CIP, ciprofloxacin; IMP, imipenem; LEV, levofloxacin; MEM, meropenem; CRO, ceftriaxone; CN, gentamicin; CAZ, ceftazidime; CTX, cefoxitin; AMK, amikacin; CT, colistin. (**b**) ML phylogeny reconstructed from 826 non-recombinant SNPs identified in 9 ST16 outbreak strains and 36 global ST16 *K. pneumoniae *. An ST412 isolate was used as an outgroup to root the tree. The coloured circles at terminal leaves highlight the outbreak isolates (red) and environmental isolates (cyan). The tip labels highlighted with grey backgrounds indicate ST16 isolates from Vietnam. The stars show bootstrap support values ≥80 % on internal nodes, with larger stars showing higher bootstrap values. Bar, the number of nucleotide substitutions per site. The heat map shows the presence (blue colour) or absence (grey colour) of resistance genes.

The three *K. pneumoniae* isolates from outbreak one displayed identical AMR profiles, showing resistance to ampicillin, levofloxacin, ciprofloxacin, co-trimoxazole, imipenem, meropenem (MIC: 12–>32 µg ml^−1^) and colistin (MIC: 8–16 µg ml^−1^), with susceptibility to amikacin, gentamicin, ceftazidime and ceftriaxone. None of the isolates from outbreak one were extended-spectrum β-lactamase positive. The five *K. pneumoniae* isolates from outbreak two also exhibited similar AMR profiles; however, the outbreak two AMR profile was distinct from that of the outbreak one isolates. Compared to the outbreak one isolates, outbreak two isolates were also resistant to ampicillin, levofloxacin, ciprofloxacin, co-trimoxazole, imipenem and meropenem (MIC: 4–24 µg ml^−1^) and susceptible to amikacin (MIC: 2–4 µg ml^−1^), yet they were additionally resistant to gentamicin (MIC: >16 µg ml^−1^), ceftazidime (MIC: >64 µg ml^−1^) and ceftriaxone (MIC: >256 µg ml^−1^), and susceptible to colistin (MIC: 0.09–0.13 µg ml^−1^). The AMR profiles of the two *K. pneumoniae* environmental isolates obtained on the VA ward during outbreak two were identical to those of the outbreak two patient isolates, with the exception of patient three involved in this outbreak (P3_O2; Fig. 2a). All *K. pneumoniae* outbreak and environmental isolates from outbreak two were extended-spectrum β-lactamase positive.

### Phylogenetics and AMR/virulence genetic determinants

Phylogenetic reconstruction of all *K. pneumoniae* isolates (outbreak one, three isolates; outbreak two, five isolates; environmental samples, two isolates) demonstrated that all except one belonged to the KpI phylogenetic group (* K. pneumoniae *). The exception was one microbiologically suspected *K. pneumoniae* isolate from outbreak two (P3_O2); this isolate clustered within the KpII phylogenetic group, which is defined as *Klebsiella quasipneumoniae* although it is biochemically indistinguishable from *K. pneumoniae* [22, 48] (Fig. S1). Notably, all KpI isolates belonged to a single ST (ST16), whilst the KpII isolate was ST446.

Aiming to investigate the potential origins and transmission of *K. pneumoniae* ST16 outbreak isolates at a larger scale, a second phylogeny was reconstructed from our 9 ST16 isolates together with a collection of all publicly available *K. pneumoniae* ST16 (36 isolates). Phylogenetic analysis showed that the three *K. pneumoniae* isolates from outbreak one formed a tight, well-supported cluster with only one SNP difference among the three isolates (Fig. 2b). These data confirm that outbreak one was likely caused by a single strain that had been transmitted among the three patients. Additionally, we found that outbreak one isolates shared a most recent common ancestor with previously described isolates from other hospitals in Ho Chi Minh City in 2015 and 2019. Among the four ST16 isolates from outbreak two, three of them formed a cluster with only 1 SNP difference, whilst the other (P4_O2) was more distantly related (46 SNPs difference) (Fig. 2b). Therefore, our data revealed that outbreak two was caused by more than one strain, with one case cluster (*n*=3) identified amongst the five *K. pneumoniae* detected in patients in this ward during the outbreak period. Notably, the outbreak two isolates also clustered with two isolates previously sampled in the same hospital between 2013 and 2014. Furthermore, we found that the two ST16 environmental isolates identified from a hospital bed and blood pressure cuff were identical and clustered tightly with the outbreak two case cluster, with only one SNP difference (Fig. 2b). Despite the fact that the two outbreaks were caused by a single genotype of ST16, our fine-scale phylogenetic analysis demonstrated that outbreak one and outbreak two isolates belonged to two separate lineages, indicative of two distinct but temporally overlapping outbreaks.

Regarding the AMR gene profile, all ST16 outbreak one isolates carried an identical set of acquired AMR genes including *qnrS*, *rmtB*, *mphA* and *bla*_OXA-181_ conferring resistance to quinolones, aminoglycosides, macrolides and carbapenems. Hybrid *de novo* assembly using Nanopore long-read sequences and Illumina data from an outbreak one isolate resulted in a full-length IncFII plasmid of 87 kb (accession number: MT635909) carrying the carbapenem-resistance gene *bla*_OXA-181_, downstream of an insertion sequence, IS*kpn19* (Fig. S2). This plasmid harboured self-transfer modules and was most similar to plasmid pSECR18-2374C from a *K. pneumoniae* isolate described from South Korea in 2019 (accession number: CP041930.1) (coverage 80 %, identity 99 %, blastn search). Compared to the outbreak one isolates, the four ST16 outbreak two isolates carried a higher number of acquired AMR genes. More specifically, three ST16 isolates within the case cluster carried an extensive array of resistance genes, including *arr3*, *catA*, *aadA16*, *rmtB*, *sulI*, *mphA*, *bla*_TEM-1_, *bla*_CTX-M-15_, *dfrA*, *qnrS*, *qnrB*, *tetA* and *bla*_OXA-181_ associated with resistance to rifampicin, chloramphenicol, aminoglycosides, macrolides, third-generation cephalosporins, co-trimoxazole, fluoroquinolones, tetracyclines and carbapenems. The *bla*_OXA-181_ gene from these isolates was also found on an IncFII plasmid that shared similar genetic structure with the IncFII plasmid from the outbreak one isolates (coverage 96 %, identity 99 %). In addition, the remaining ST16 isolate from outbreak two carried *aac3-IIa*, *aac6-ib-cr*, *catB*, *sulII*, *bla*_TEM-1_, *bla*_CTX-M-15_, *dfrA*, *qnrB*, *tetA*, *strAB* and *bla*_OXA-48_ associated with resistance to aminoglycosides, chloramphenicol, third-generation cephalosporins, co-trimoxazole, fluoroquinolones, tetracyclines and carbapenems. The *bla*_OXA-48_ gene was found on an IncL plasmid of about 60 kb and was most similar to the plasmid pOXA48 (GenBank accession number: JN626286) (coverage 90 %, identity 99 %). All ST16 isolates carried chromosomal mutations on *gyrA* (S83F, D87N) and *parC* (E84K), which confer resistance to fluoroquinolones. Furthermore, all *K. pneumoniae* isolates from outbreak and environmental samples carried genes encoding a yersiniabactin siderophore. However, the yersiniabactin STs differed between the outbreak one isolates (YbST174) and outbreak two isolates (YbST183).

### Colistin-resistance mechanism

All three ST16 outbreak one isolates were highly resistant to colistin (MIC: 8–16 µg ml^−1^). However, we did not detect any chromosomal mutations in the TCSs including PhoQ/P, PmrA/B and CcrA/B or the plasmid-mediated *mcr* gene. Aiming to characterize the colistin-resistance mechanism, we performed long-read Nanopore sequencing and further analyses. We found that colistin resistance was due to disruption of the *mgrB* gene by an IS*L3*-like insertion sequence at position +12 in the coding region [31]. This IS*L3*-like element was 1304 bp in length, targeted an AT-rich region (8 bp) on the *mgrB* gene and resulted in duplication at the target site during transposition (Fig. S3). This IS*L3*-like element was additionally found in two other chromosomal regions, including the intergenic region between the *cmoB* (RNA U34 carboxymethyltransferase) and *cutC* genes (copper homeostasis protein) and the gene encoding a sensor domain-containing diguanylate cyclase. In addition, one copy of the IS*L3*-like element was also found on the *bla*_OXA-181_-carrying IncFII plasmid (positions: 40709–41911).

## Discussion

*K. pneumoniae* has emerged as one of the most common causes of HAIs in low- and middle-income countries, and is associated with high morbidity and mortality [14, 58, 59]. HAIs caused by this clinically important pathogen are becoming difficult to treat due to the emergence of multidrug resistance, especially resistance to last-line drugs such as carbapenems and colistin. Nosocomial outbreaks caused by carbapenem-resistant *K. pneumoniae* are frequently reported worldwide but have rarely been reported in Vietnam, possibly due to a lack of hospital surveillance, diagnostics, and a high-resolution genotyping method for the characterization and investigation of outbreaks. In this study, two distinct but temporally overlapping nosocomial outbreaks with high mortality occurred in two different wards of a tertiary hospital in Vietnam, were promptly identified by clinical microbiologists through routine surveillance, and were rapidly characterized and contained through cutting-edge laboratory investigation and the deployment of timely hospital infection control measures. Given that the two *K. pneumoniae* outbreaks were initially identified based solely on an increase in carbapenem resistance identified in the wards, future outbreaks will be more difficult to detect if carbapenem resistance becomes widespread, highlighting the need for coordinated efforts between clinical microbiologists, molecular biologists and infection control teams to provide evidence-based guidance for the containment and prevention of outbreaks in hospital settings.

This study highlights the value of using high-throughput genome sequencing for the characterization of hospital-based outbreaks. By using WGS, we were able to delineate the genetic relatedness of *K. pneumoniae* isolates from the two outbreaks and environmental samples, and identify two case clusters corresponding to the two separate outbreaks. Furthermore, we found *K. pneumoniae* isolates identified from a hospital bed and blood pressure cuff during outbreak two that were genetically linked to the outbreak two case cluster, suggesting such high-touch equipment may be an important source of *K. pneumoniae* maintenance in the hospital. Additionally, during outbreak one, carbapenem- and colistin-resistant *K. pneumoniae* isolates were also identified from various pieces of hospital equipment, such as patient monitors, ventilators, medical trolleys and bedrails. Though these isolates were not genome sequenced, they were likely linked to the outbreak isolates given their shared resistance patterns against carbapenems and colistin. Previous studies have also shown that bedrails, bedside items and bed surfaces are among the most-contaminated surfaces [60–62], while blood pressure cuffs are a known vehicle of bacterial transmission, particularly in ICUs [63]. Regular and appropriate cleaning of hospital surfaces and improved personal hand hygiene are strongly advised to reduce colonization of deadly resistant bacteria and prevent nosocomial infections. Further study is also warranted to identify the most common contaminated surfaces in hospital environments, and evaluate the impact of different decontamination approaches on the colonization and further spread of nosocomial pathogens.

Here, we found that the two nosocomial outbreaks were caused by two distinct lineages of a carbapenem-resistant *K. pneumoniae* ST16 clone. Notably, these ST16 outbreak isolates also clustered tightly with carbapenem-susceptible ST16 isolates previously described from the same hospital in 2013 and 2014, as well as other hospitals in the south of Vietnam, suggesting that this particular clone may have been maintained in our hospital or other hospitals in Vietnam. The frequent transfer of severely ill patients across different wards in the same hospital and between different hospitals is likely an important factor contributing to the transmission of this pathogen. We observed that ST16 outbreak isolates have gained extensive resistance to almost all drugs normally used for the treatment of *K. pneumoniae* infections, which might result from long-term adaptation to antimicrobial usage in hospital settings. The acquisition of extensive drug resistance in ST16 outbreak isolates likely contributed to its high mortality (2/3 and 4/5 patients from outbreak one and outbreak two died, respectively). Outbreaks caused by *K. pneumoniae* are commonly caused by high-risk carbapenem-resistant clones within CC258, including ST258, ST11 and other closely related STs [18, 64–67]. Overall, *K. pneumoniae* ST16 is not a commonly reported clone; however, recent studies suggest that ST16 is an emerging international lineage that frequently shows resistance to carbapenems, and has been responsible for several nosocomial outbreaks with mortality rates reaching up to 75 % [68, 69]. Currently, little is known about the population structure and epidemiological factors facilitating the spread of this clone. Therefore, further investigation of retrospective collections at local hospitals and development of hospital-network-based surveillance should be prioritized to track the evolution and transmission of the ST16 clone and other epidemiologically and clinically important high-risk clones, in order to guide proper clinical practices and improve infection control strategies.

We found two different plasmid structures carrying carbapenem resistance genes among the ST16 outbreak isolates, including IncFII/*bla*_OXA-181_ and IncL/*bla*_OXA-48_. *bla*_OXA-181_, a variant of *bla*_OXA-48_ with 4 amino acid substitutions, was first reported in India and subsequently found in the UK, The Netherlands, France, New Zealand, Oman and Singapore [70]. In Vietnam, previous studies involving molecular typing of carbapenem-resistance genes in *K. pneumoniae* did not find *bla*_OXA-181_, but instead identified *bla*_KPC-2_, *bla*_NDM-1_, *bla*_NDM-4_ and *bla*_OXA-48_ [41, 71]. Apart from *K. pneumoniae *, *bla*_OXA-181_ has been sporadically found in other Gram-negative bacteria such as *Escherichia coli* [72], *Enterobacter cloacae* [73]*, Citrobacter freundii* [74] and *Morganella morganii* [75]. The fact that *K. pneumoniae* ST16 isolates from the two outbreaks belonged to distinct lineages and both also harboured the transferable IncF/*bla*_OXA-181_ plasmid suggests that this plasmid was acquired independently by phylogenetically unrelated isolates. The reservoir and biology of this carbapenem-resistance plasmid warrants further investigation, as such plasmids can spread efficiently to other *K. pneumoniae* clones or members of the *Enterobacteriaceae* through horizontal transfer.

In this study, we also investigated the colistin resistance mechanism in the ST16 outbreak one isolates. Few studies have reported colistin-resistant *K. pneumoniae* in Vietnam, and the mechanisms of this resistance remain poorly characterized [41, 71]. Using long-read Nanopore sequencing, we found that disruption of the *mgrB* gene by an IS*L3*-like element resulted in the colistin resistance; disruption of the *mgrB* gene by insertion sequences is a commonly reported mechanism of this resistance [31], and such resistance via *mgrB* gene inactivation has been found to occur during the course of colistin treatment [73]. Notably, we found three additional copies of this IS*L3*-like element, two of which were inserted in chromosomal regions and the other was identified in the IncF/*bla*_OXA-181_ plasmid. The presence of the IS*L3*-like element on the carbapenem resistance-inducing IncF/*bla*_OXA-181_ plasmid is concerning, as the presence of this plasmid may give rise to colistin resistance during the course of treatment in individual patients.

Here, we have described two nosocomial outbreaks with high mortality caused by carbapenem-resistant *K. pneumoniae* ST16 in two different wards of a tertiary hospital in southern Vietnam, and found that two distinct XDR lineages of ST16 were responsible for the two outbreaks. *K. pneumoniae* outbreak isolates were also detected from high-touch hospital surfaces during the outbreaks. Carbapenem resistance was mediated by the transferable IncF/*bla*_OXA-181_ plasmid carrying an IS*L3*-like element, which can induce colistin resistance. Routine microbiological and molecular surveillance, and strengthening of hospital infection control and prevention measures are needed to identify outbreaks and prevent the transmission of nosocomial pathogens.

## Supplementary Data

Supplementary material 1

Supplementary material 2
