# Supplementary material for: Emerging carbapenem-resistant Klebsiella pneumoniae sequence type 16 causing multiple outbreaks in a tertiary hospital in southern Vietnam
Source: Microb Genom. 2021 Feb 10;7(3):mgen000519. doi: 10.1099/mgen.0.000519 (PMC8190610; doi:10.1099/mgen.0.000519)
Supplement: Supplementary material 1 [file mgen-7-0519-s001.pdf]

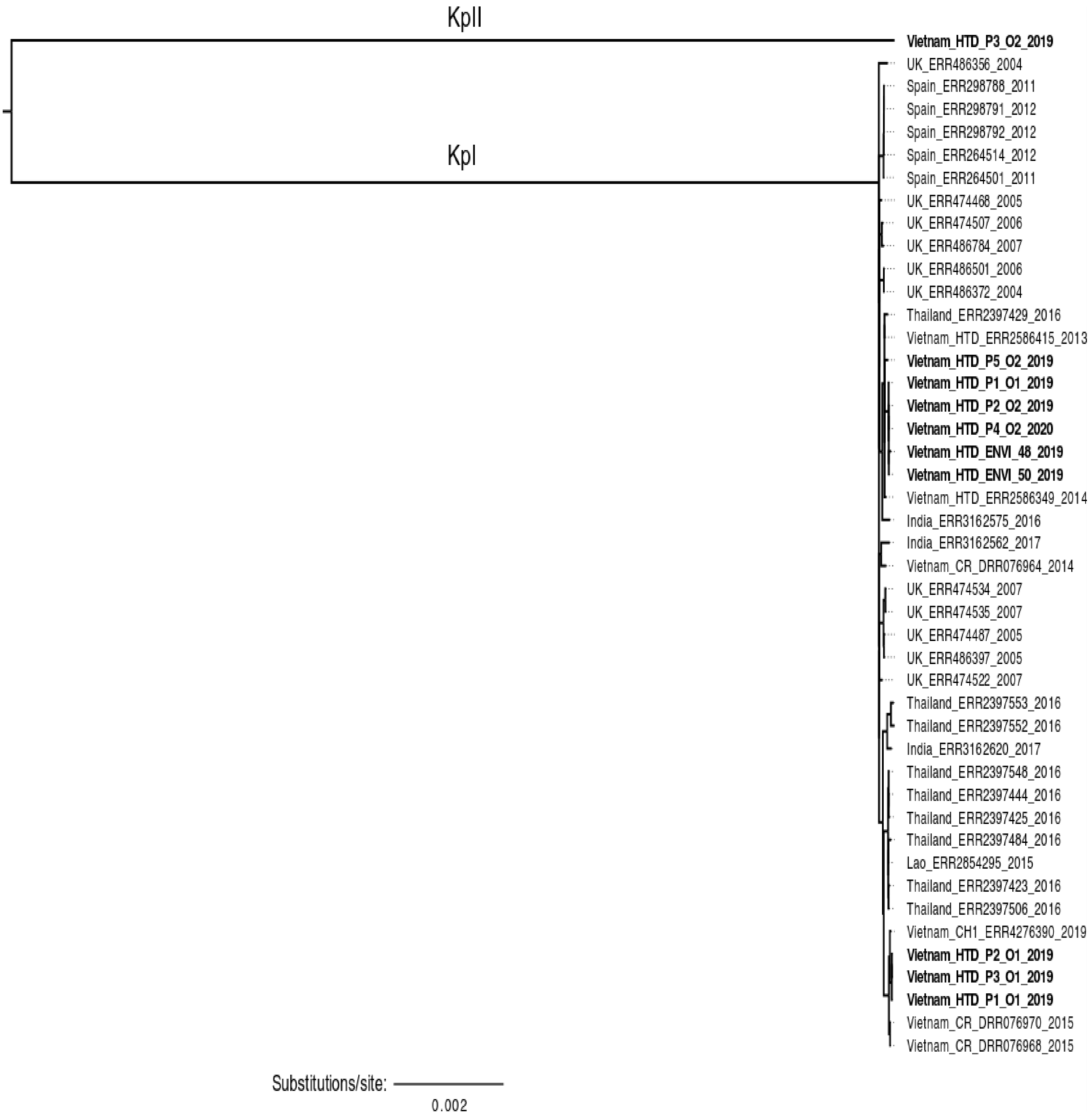

Figure S1. Phylogenetic relatedness between one ST446 outbreak 2 isolate (KpII) and the remaining ST16 outbreak and environmental isolates (KpI).
Midpoint rooted phylogeny estimated from the alignment of 12,190 core SNPs identified from on ST446 (*Klebsiella quasipneumoniae*) isolate and nine ST16 isolates (*Klebsiella pneumoniae*) in our study, together with a global collection of 36 ST16 *K. pneumoniae*. The bolded tips highlight the isolates from our study. The scale bar represents nucleotide substitutions per site.

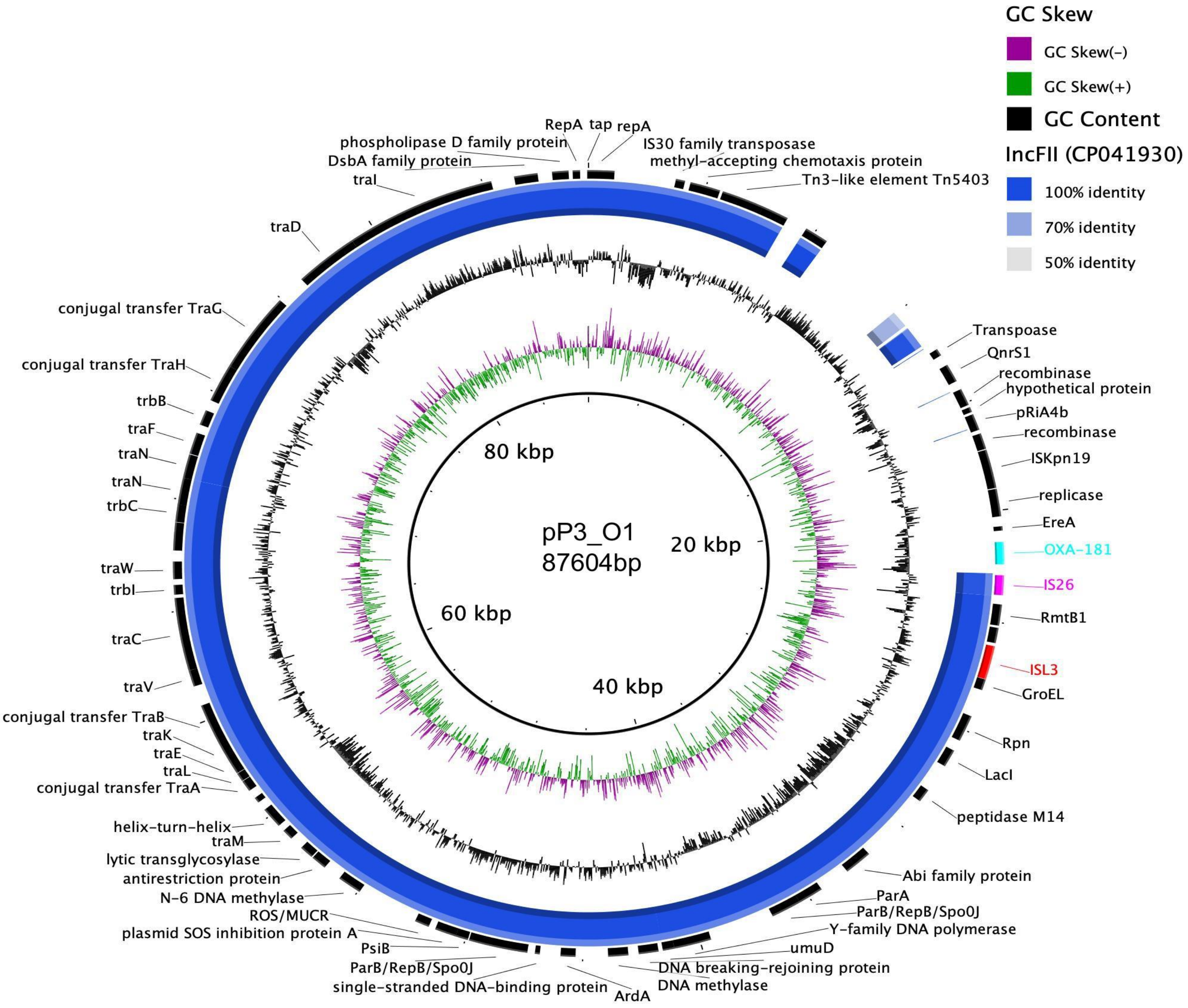

Figure S2. Genetic structure of *bla*$_{OXA181}$-carrying plasmid pP3_O1.
BLASTN comparison between the *bla*$_{OXA181}$-carrying plasmid pP3_O1 (central ring) identified from a carbapenem resistant ST16 outbreak isolate and a reference plasmid from GenBank (Accession number: CP041930.1). The outermost ring indicates the gene annotations of the pP3_O1 plasmid. The ISL3-like element is highlighted in red. Cyan and pink colors indicate the *bla*$_{OXA181}$ gene and IS*26*, respectively.

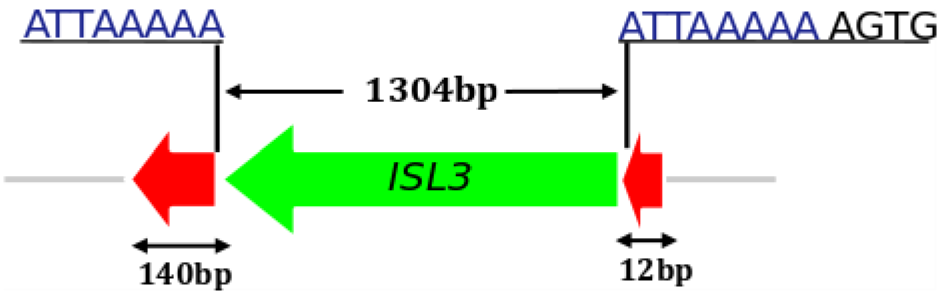

Figure S3. Schematic representation of the insertion event occurring in the *mgrB* gene.
The duplicated sequences are shown in blue (upstream and downstream of the *mgrB* gene). Red arrows show the interruption of the *mgrB*gene due to the insertion of the ISL3-like element (green).
